# Spigelian hernia in the elderly

**DOI:** 10.1186/1471-2318-11-S1-A20

**Published:** 2011-08-24

**Authors:** R M Giamattei, L Butuc, F La Rocca, G Viola, A Mercogliano, G Fatigati, A Martino

**Affiliations:** 1 Dipartimeno di Chirurgia Generale e Specialistica, Casa di cura Andrea Grimaldi – San Giorgio a Cremano, Italy

## 

The Spigelian hernia or lateral ventral hernia is a rare clinical entity, characterized by a defect in the Seminular line. The etiology is mainly based on the anatomical features of the Spigelian aponeurosis and all those conditions that lead to increased intra-abdominal pressure (COPD, obesity, chronic constipation), typical in the elderly.

In our study, we report two cases of Spigelian hernia which appear in two patients, a 75-year-old female and a 71-year-old male, both had associated comorbidities. The female patient, suffering from hypertension and a history of diverticular disease which presents a strangulated hernia of the left abdominal wall, so, in the presence of intestinal sub-occlusion sintomatology, required emergency treatment. The second patient, suffering from hypertension, kidney stones and benign prostatic hyperplasia, reported abdominal discomfort with intense swelling, predominantly on the left of the abdominal examination. The second patient did not present complication and elective treatment was performed. The surgical technique adopted in both cases was the placement of hernioplasty with polypropylene mesh, a technique that allows a short hospital stay and early rehabilitation for the patient.

